# Effect of COVID-19 infection on psychological aspects of pre-schooler children: a cross-sectional study

**DOI:** 10.1186/s43045-022-00207-y

**Published:** 2022-06-03

**Authors:** Gellan K. Ahmed, Sayed Mostafa, Khaled Elbeh, Hamdy M. Gomaa, Saeed Soliman

**Affiliations:** 1 Department of Neurology and Psychiatry, Faculty of Medicine, Assiut University, Assiut, Egypt; 2 Department of Child & Adolescent Psychiatry, Institute of Psychiatry, Psychology & Neuroscience, King’s College London, London, SE5 8AF UK; 3 Department of Family Medicine, Faculty of Medicine, Assiut University, Assiut, Egypt; 4 Department of Family Medicine, Faculty of Medicine, Cairo University, Cairo, Egypt

**Keywords:** COVID-19, Children, Psychiatric comorbidity

## Abstract

**Background:**

Coronavirus disease 2019 (COVID-19) had a tremendous effect on individual’s lives worldwide. The pandemic’s significant socioecological impact is one of the many burdens children confront in the current crises. As a result, this study was designed to determine the psychological impacts of the COVID-19 pandemic on preschoolers, particularly the consequences of COVID-19 infection. This study involved 138 children aged 2–5.11 years old who were classified into two groups based on their COVID-19 infection history, which was documented via a PCR test. All participants were assessed by the Socioeconomic Scale and The Children’s Behavior Checklist (CBCL).

**Results:**

COVID-19 infection was found in 21.7% of the children who participated in this study. Furthermore, children with COVID-19 had a higher percentage of clinical rating on the CBCL Profile of DSM-5 scales for affective problems (13.3 vs. 7.4%), anxiety problems (13.3 vs. 9.3%), pervasive developmental problems (20 vs. 13%), and oppositional defiant problems (6.7 vs. 5.6%) than children without COVID-19. Anxiety and somatic problems had a positive correlation with the impact of the COVID-19 pandemic on the lives of children.

**Conclusions:**

Children infected with COVID-19 were more likely to have psychological issues, such as affective disorders, anxiety problems, pervasive developmental problems, and oppositional defiant problems. These psychological issues had a relationship with the impact of the COVID-19 pandemic on the lives of children.

## Background

Coronavirus disease 2019 (COVID-19) had a tremendous effect on individual’s lives worldwide. Isolation, communication limitations, and economic collapse all significantly affected countries’ psychosocial environments. The circumstance has a significant effect on kids, teenagers, and families. Schools and kindergartens have been shut down, social connections are strictly restricted, and recreational activities outside the home have been canceled. Parents are expected to work from home while still supporting their children’s homeschooling. In the absence of other family members or social support systems, external assistance is no longer available. While COVID-19 has been a major issue and long-term impact on health and mental health [1], the economy has been in decline, with increasing unemployment rates in all affected countries. Children, teenagers, and families are under a lot of stress, which could lead to mental health issues and violence [2]

The pandemic’s significant socioecological impact is one of the many burdens children confront in the current crises. Children’s environments are influenced by various perspectives, including those of their families and communities and their viewpoints themselves [3].

Since the pandemic was revealed, essential services, such as nursing, schools, and basic medical support, have been disrupted or restricted at the neighborhood level [4]. Notably, child services and current programs of assistance or monitoring protection have been affected and suspended [5]. The lack of these essential services can be terrible for children and families in trouble. Furthermore, leisure activities have been restricted: in most countries, public playgrounds are closed and social group activities are prohibited [4].

Inadequate attention to the mental health of children and teenagers can lead to long-term mental problems, which can affect a person’s ability to lead a healthy and productive life [6]. As a result, mental health problems in children and adolescents frequently have long-term severe negative consequences [7–9].

Early diagnosis and treatment of these problems are in everyone’s best interest, especially for kids, teens, families, and societies [10–12]. Community burden, measurement, and the survey’s planning and execution were all significant ways in which epidemiology might help better understand children’s and adolescents’ mental health [13].

As a result, this study was designed to determine the psychological impacts of the COVID-19 pandemic on preschoolers, particularly the consequences of COVID-19 infection.

## Methods

### Participants and procedures

A cross-sectional study was conducted from February 2021 to May 2021. In this study, 138 children aged 2–5.11 years were selected from three Assiut government kindergarten’s facilities. Children were selected from three Assiut government kindergartens’ facilities to ensure diversity of sociodemographic background as 3 kindergartens were from 3 different areas of the city (from the east, west, and centers of the city). Students were permitted to go to school 2 days per week at this time. The sample size was calculated using the statcalc program of EPI-info version 7.2 using population survey or descriptive observational study calculation, according to the proportion of COVID-19 infection among preschool children of 3.8% [14]. The acceptable margin of error was 5%, confidence level 95%, and design effect 1%. The minimum required sample size should be 57 patients. The recruited children were allowed to attend their kindergarten after their COVID-19 infection for at least 2 weeks. We obtained approval from the managers of the kindergartens’ facilities to post an invitation using Google form on the website of the kindergarten’s facilities requesting parents to join in the evaluation of their kids for our research. At Assiut University’s Child Psychiatry Clinic, we conducted interviews with the parents and children who accepted our participation request. Furthermore, the participants were split into two groups: those with a history of COVID-19 infection (*N* = 30) as evidenced by a positive polymerase chain reaction (PCR) report and those without a history of COVID-19 infection (*N* = 108). Participants with mental disorders, neurological or medical problems, or an intelligence quotient of less than 70 were excluded from the study.

### Tools

A semi-structured interview was conducted by the researchers, which provided data on the participants’ full mental and medical history at the start of the study. Parents were asked about their children’s past COVID-19 infection, which was confirmed by a PCR report, and the length of time since the diagnosis.

Age, gender, birth order, number of children, delivery type and problems, speech and motor development delays, and family history of psychiatric disorders were among the sociodemographic data obtained.

### The impact of the COVID-19 pandemic on the lives of children

This was gathered by some questions, such as “Is there a member of the family (first-degree relative) who has COVID-19 infection?,” “Is there anyone in the family (first-degree relative) who has died of COVID-19 infection?,” “Were there school issues (academic) as a result of COVID-19 infection?,” “Did COVID-19 infection cause social issues (peers)?,” “Did COVID-19 infection cause problems in parent–child relationships?,” “Did the household expenses increase as a result of COVID-19 infection?,” and Has COVID-19 infection resulted in a decrease in household income?.”

The Socioeconomic Scale [15] is a tool used to determine socioeconomic burdens and social classes. It also considers four essential factors: the educational levels of the father and mother, their individual employment, the family’s overall income, and the family’s standard of living.

The Children’s Behavior Checklist (CBCL) [16] is a 100-item parent-reported questionnaire meant to track preschoolers’ troublesome behaviors. The responses are rated based on the child’s behavior in the past 6 months.

### Statistical analysis

A statistical package for the social sciences was used for all statistical analyses (version 26). Frequencies and percentages were used to express descriptive data. To investigate categorical variables, the chi-square test was performed. To analyze quantitative variables and determine differences in mean values between the two groups, the independent *t* test was performed. To investigate the relationship between several variables, the spearman correlation was applied. A point-biserial correlation coefficient was used to have a correlation for a dichotomous categorical variable and a continuous variable. The statistical significance was determined by *P*-values of less than 0.05.

## Results

### Data on socioeconomics

There was no significant difference between the groups under study regarding sociodemographic data. This study comprised 138 preschool-aged children. COVID-19 infection was found in 21.7% of the children who participated in this study. More than half of the participants were females (56.5%); however, more than half of the participants who had COVID-19 were males (60%). Regarding birth order, most participants were born first. Moreover, most participants who underwent Cesarean section had no postpartum complications, had normal speech and motor development, no family history of psychiatric illnesses, and a socioeconomic standing in the middle (see Table 1).

**Table 1:** Sociodemographic data among studied groups

Variables	Children who had not COVID-19 (*n*=108)	Children who had COVID-19 (*N*=30)	Total participants (*N*=138)	T value	Chi-square value	*P*-value
**Age (years) (mean±SD)**	4.1±1.01	4.2±0.76	4.13±0.96	0.19	-	0.65
**Gender**						
Males	42 (38.9%)	18 (60%)	60 (43.5%)	-	4.25	0.06
Females	66 (61.1%)	12 (40%)	78 (56.5%)			
**Order of birth**						
First	60 (55.6%)	16 (53.3%)	76 (55.1%)	-	0.43	0.8
Second	30 (27.8%)	10 (33.3%)	40 (29%)			
Third or more	18 (16.7%)	4 (13.3%)	22 (15.9%)			
**Number of children**						
Only child	16 (14.8%)	6 (53.3%)	22 (15.9%)	-	3.7	0.15
Two	42 (38.9%)	16 (53.3%)	58 (42%)			
Three or more	50 (46.3%)	8 (26.7%)	58 (42%)			
**Delivery type**						
Normal	18 (16.7%)	4 (13.3%)	22 (15.9%)	-	0.19	0.78
Caesarean section	90 (83.3%)	26 (86.7%)	116 (84.1%)			
**Post-partum problems for children**						
**Incubator admission**						
Yes	18 (16.7%)	2 (6.7%)	20 (14.5%)	-	1.8	0.49
No	90 (83.3%)	28 (93.3%)	118 (85.5%)			
**Speech development**						
Delay	30 (27.8%)	8 (26.7%)	38 (27.5%)	-	0.015	0.9
Normal	78 (72.2%)	22 (73.3%)	100 (72.5%)			
**Motor development**						
Delay	4 (3.7%)	2 (6.7%)	6 (4.3%)	-	0.49	0.6
Normal	104 (96.3%)	28 (93.3%)	132 (95.7%)			
**Family history of psychiatry disorders**						
Yes	10 (9.3%)	2 (6.7%)	12 (8.7%)	-	0.19	0.9
No	98 (90.7%)	28 (93.3%)	126 (91.3%)			
**Socioeconomic level**	219.82±37.91	217.34±36.9	219.28±37.58	0.101	-	0.75
Low	12 (11.1%)	6 (20%)	18 (13%)	-	1.7	0.43
Middle	86 (79.6%)	22 (73.3%)	108 (78.3%)			
High	10 ( 9.3%)	2 (6.7%)	12 (8.7%)			

*Significant *P*-value

### The impact of the COVID-19 pandemic on the lives of children

A child with COVID-19 infection was significantly more impacted in their life by the pandemic in terms of family members infected or dying from it, and the effects it had on their educational, social, and parental issues increased expenses and decreased income. The mean of the duration of disease was 1.4 months (see Table 2).

**Table 2:** Association between COVID-19 infection status and areas of children’s life

Variables	Children who had not COVID-19 (*n*=108)	Children who had COVID19 (*N*=30)	Total participants (*N*=138)	Chi-square value	*P*-value
Did any one of the family (first relative degree) have diagnosis as COVID-19 infection.	30 (27.8%)	28 (93.3%)	58 (42%)	41.4	0.001*
Did any one of the family (first relative degree) die from COVID-19 infection.	0 (0%)	6 (20%)	6 (4.3%)	22.5	0.001*
School problems (academic) due to COVID-19	14 (13%)	10 (33.3%)	24 (17.4%)	6.7	0.014*
Social problems (peers) due to COVID-19	28 (25.9%)	18 (60%)	46 (33.3%)	12.26	0.0001*
Problems in parent relationship due to COVID-19	6 (5.6%)	8 (26.7%)	14 (10.1%)	11.4	0.002*
Increase Family expenses due to COVID-19	6 (5.6%)	7 (23.3%)	13 (9.4%)	8.6	0.004*
Decrease family income due to COVID-19	14 (13%)	8 (26.7%)	22 (15.9%)	3.2	0.18

*Significant p value

### CBCL outcome

According to CBCL scores, the percentage of children with clinical ratings of syndrome profiles during the COVID-19 pandemic was 18.8% for withdrawal, 7.2% for somatic complaints, 14.5% for anxious/depressed, 5.8% for sleep problems, 4.3% for emotionally reactive behavior, 5.8% for aggressive behavior, 8.7% for affective problems, 10.1% for anxiety problems, 15.9% for pervasive developmental problems, and 5.8% for aggressive behavior.

In terms of somatic complaints, sleep problems, attention problems, oppositional defiant behavior, externalizing problems, and total problems, significant statistical differences were observed between the groups under study. A higher percentage of clinical rating was found in children who had COVID-19 than in children who did not have COVID-19 in the zones of withdrawal (26.7 vs. 16.7%, respectively), somatic complaints (26.7 vs. 1.9%, respectively), anxiety/depression (20 vs. 13%, respectively), sleep problems (13.3 vs. 3.7%, respectively), emotional reactivity (6.7 vs. 3.7%, respectively), and aggression (6.7 vs. 5.6%).

Furthermore, children with COVID-19 had a higher percentage of clinical rating on the CBCL Profile of DSM-5 scales for affective problems (13.3 vs. 7.4%), anxiety problems (13.3 vs. 9.3%), pervasive developmental problems (20 vs. 13%), and oppositional defiant problems (6.7 vs. 5.6%) than children without COVID-19 (see Tables 3 and 4).

**Table 3:** CBCL profile of syndromes of studied group

Profile of syndromes		Children who had not COVID-19 (*n*=108)	Children who had COVID-19 (*N*=30)	Total participants (*N*=138)	Chi-square value	*P*-value
Withdrawn	Normal	74 (68.5%)	*20 (66.7%)*	*94 (68.1%)*	2.45	0.28
	Borderline	16 (14.8%)	2 (6.7%)	18 (13%)		
	Clinical rating	18 (16.7%)	8 (26.7%)	26 (18.8%)		
Somatic complaints	Normal	100 (92.6%)	18 (60%)	118 (85.5%)	*24.82*	<0.000*
	Borderline	6 (5.6%)	4 (13.3%)	10 (7.2%)		
	Clinical rating	2 (1.9%)	8 (26.7%)	10 (7.2%)		
Anxious/depressed	Normal	80 (74.1%)	*18 (60%)*	*98 (71%)*	*2.25*	*0.33*
	Borderline	14 (13%)	6 (20%)	20 (14.5%)		
	Clinical rating	14 (13%)	6 (20%)	20 (14.5%)		
Sleep problems	Normal	102 (94.4%)	24 (80%)	*126 (91.3%)*	*6.17*	0.04*
	Borderline	2 (1.9%)	2 (6.7%)	*4 (2.9%)*		
	Clinical rating	4 (3.7%)	4 (13.3%)	*8 (5.8%)*		
Attention problems	Normal	108 (100%)	26 (86.7%)	134 (97.1%)	*14.83*	0.002*
	Borderline	0 (0%)	4 (13.3%)	4 (2.9%)		
	Clinical rating	0 (0%)	0 (0%)	0 (0%)		
Emotionally reactive	Normal	82 (75.9%)	*22 (73.3%)*	*104 (75.4%)*	*0.49*	*0.78*
	Borderline	22 (20.4%)	6 (20%)	28 (20.3%)		
	Clinical rating	4 (3.7%)	2 (6.7%)	6 (4.3%)		
Aggressive behavior	Normal	94 (87%)	22 (73.3%)	116 (84.1%)	4.24	0.15
	Borderline	8 (7.4%)	6 (20%)	14 (10.1%)		
	Clinical rating	6 (5.6%)	2 (6.7%)	8 (5.8%)		
General profile of problems						
Internalizing problems	Normal	74 (68.5%)	18 (60%)	92 (66.7%)	2.64	0.26
	Borderline	10 (9.3%)	6 (20%)	16 (11.6%)		
	Clinical rating	24 (22.2%)	6 (20%)	30 (21.7%)		
Externalizing problems	Normal	90 (83.3%)	18 (60%)	108 (78.3%)	8.68	0.013*
	Borderline	6 (5.6%)	6 (20%)	12 (8.7%)		
	Clinical rating	12 (11.1%)	6 (20%)	18 (13%)		
Total problems	Normal	82 (75.9%)	14 (46.7%)	96 (69.6%)	20.26	<0.000*
	Borderline	8 (7.4%)	12 (40%)	20 (14.5%)		
	Clinical rating	18 (16.7%)	4 (13.3%)	22 (15.9%)		

*Significant *P*-value

**Table 4:** CBCL profile of DSM-5 scales of studied group

Profile of DSM-5 scales		Children who had not COVID-19 (*n*=108)	Children who had COVID-19 (*N*=30)	Total participants (*N*=138)	Chi-square value	*P*-value
Affective problems	Normal	94 (87%)	26 (86.7%)	120 (87%)	*2.6*	*0.27*
	Borderline	6 (5.6%)	0 (0%)	6 (4.3%)		
	Clinical rating	8 (7.4%)	4 (13.3%)	12 (8.7%)		
Anxiety problems	Normal	88 (81.5%)	20 (66.7%)	108 (78.3%)	*3.37*	*0.18*
	Borderline	10 (9.3%)	6 (20%)	16 (11.6%)		
	Clinical rating	10 (9.3%)	4 (13.3%)	14 (10.1%)		
Pervasive developmental problems	Normal	78 (72.2%)	22 (73.3%)	100 (72.5%)	*1.2*	*0.54*
	Borderline	14 (13%)	2 (6.7%)	16 (11.6%)		
	Clinical rating	16 (14.8%)	6 (20%)	22 (15.9%)		
Attention deficit /hyperactivity problems	Normal	102 (94.4%)	30 (100%)	132 (95.7%)	1.7	0.41
	Borderline	4 (3.7%)	0 (0%)	4 (2.9%)		
	Clinical rating	2 (1.9%)	0 (0%)	2 (1.4%)		
Oppositional defiant problems	Normal	100 (92.6%)	24 (80%)	124 (89.9%)	7.58	0.02*
	Borderline	2 (1.9%)	4 (13.3%)	6 (4.3%)		
	Clinical rating	6 (5.6%)	2 (6.7%)	8 (5.8%)		

*Significant p value

### Correlation study

In Table 5 shows the correlation for CBCL subscales and sociodemographic data and impact of COVID-19 on children. Regarding CBCL profile of syndromes, somatic problems had positive correlation school problems due to COVID-19 (*r*= 0.7, *P*-value= 0.003), social problems due to COVID-19 (*r*= 0.66, *P*-value= 0.007), and decrease family income due to COVID-19 (*r*= 0.6, *P*-value= 0.006). Sleep problems had a negative correlation with number of children (*r*= −0.5, *P*-value= 0.004).

**Table 5:** The correlation for CBCL subscales and sociodemographic data, impact of COVID-19 on children

	Age (years)		Number of children	Order of birth	one of the family have diagnosis as COVID-19 infection	one of the family die from COVID-19 infection	School problems due to COVID-19	Social problems due to COVID-19	Problems in parent relationship due to COVID-19	Increase family expenses due to COVID-19	Decrease family income due to COVID-19	Total score of socioeconomic scale
CBCL profile of syndromes												
Emotionally reactive	r	0.44	−0.05	−0.15	0.16	0.45	−0.25	0.05	0.05	−0.16	−0.07	0.17
	*P*-value	0.09	0.83	0.58	0.56	0.09	0.35	0.84	0.84	0.55	0.79	0.53
Anxious/depressed	r	0.5	0.1	−0.38	0.21	0.27	0.08	0.11	0.32	0.29	0.13	−0.14
	*P*-value	0.05	0.71	0.15	0.43	0.32	0.77	0.68	0.23	0.28	0.63	0.61
Somatic complaints	r	0.35	−0.29	−0.28	−0.32	−0.06	0.708	0.66	−0.01	0.51	0.66	−0.45
	*P*-value	0.19	0.28	0.31	0.23	0.81	0.003*	0.007*	0.95	0.05	0.006*	0.08
Withdrawn	r	0.21	−0.27	−0.03	−0.37	0.35	−0.22	0.1	−0.07	−0.15	−0.03	0.09
	*P*-value	0.45	0.33	0.89	0.16	0.19	0.42	0.71	0.8	0.58	0.9	0.72
Sleep problems	r	0.06	−0.53	−0.44	0.13	0.16	−0.1	0.000	−0.08	−0.18	−0.12	0.23
	*P*-value	0.82	0.04*	0.09	0.63	0.55	0.72	1.000	0.76	0.51	0.66	0.4
Attention problems	r	0.43	−0.05	−0.35	0.1	0.78	0.09	0.35	0.42	0.21	0.28	−0.22
	*P*-value	0.1	0.86	0.19	0.71	0.001*	0.73	0.19	0.11	0.44	0.29	0.41
Aggressive behavior	r	0.05	0.38	0.34	0.16	0.07	0.25	0.21	0.36	0.09	0.14	−0.12
	*P*-value	0.84	0.15	0.2	0.56	0.78	0.35	0.43	0.18	0.74	0.59	0.66
Internalizing problems	r	0.15	−0.27	−0.15	−0.32	0.27	−0.11	0.14	−0.01	−0.14	0.000	-0.01
	*P*-value	0.58	0.31	0.57	0.23	0.32	0.68	0.6	0.95	0.59	1.000	0.95
Externalizing problems	r	−0.20	−0.06	0.29	−0.32	-0.06	0.09	0.14	0.08	−0.06	0.03	0.14
	*P*-value	0.46	0.8	0.28	0.23	0.81	0.72	0.6	0.76	0.81	0.9	0.61
Total problems	r	0.05	−0.3	−0.39	0.28	0.13	−0.27	−0.01	0.42	0.08	−0.06	−0.09
	*P*-value	0.86	0.26	0.14	0.3	0.63	0.32	0.95	0.11	0.77	0.81	0.74
CBCL profile of DSM-5 scales												
Affective problems	r	0.14	−0.05	−0.35	0.1	0.29	0.45	0.35	0.32	0.21	0.28	-0.52
	*P*-value	0.6	0.86	0.19	0.71	0.28	0.09	0.19	0.24	0.44	0.29	0.045*
Anxiety problems	r	0.21	−0.09	−0.39	0.18	0.35	0.54	0.64	0.46	0.54	0.66	−0.29
	*P*-value	0.45	0.74	0.14	0.50	0.19	0.035*	0.01*	0.08	0.034*	0.007*	0.28
Pervasive developmental problems	r	0.44	0.38	0.09	0.16	0.82	−0.07	0.05	0.05	−0.16	−0.07	0.1
	*P*-value	0.09	0.15	0.73	0.56	0.000*	0.79	0.84	0.84	0.55	0.79	0.71
Oppositional defiant problems	r	0.31	−0.04	−0.02	0.13	0.16	−0.1	0.000	−0.08	−0.18	−0.12	0.19
	*P*-value	0.26	0.88	0.94	0.63	0.55	0.72	1.000	0.76	0.51	0.66	0.49

Regarding CBCL profile of DSM-5 scales, anxiety problems had a positive correlation with school problems due to COVID-19 (*r*= 0.5, *P*-value= 0.035), social problems due to COVID-19 (*r*= 0.6, *P*-value= 0.01), increase family expenses due to COVID-19 (*r*= 0.5, *P*-value= 0.034), and decrease family income due to COVID-19 (*r*= 0.6, *P*-value= 0.007).

Having died family member from COVID-19 infection had positive correlation with attention problems (*r*= 0.7, *P*-value= 0.0001) and pervasive developmental problems (*r*= 0.8, *P*-value= 0.0001). Total score of socioeconomic scale had negative correlation with affective problems (*r*= −0.52, *P*-value= 0.045).

## Discussion

To our knowledge, this is the first study that has assessed the psychological effects of COVID-19 infection on preschoolers. COVID-19 infection was found in 21.7% of the children in this study. Children who had COVID-19 infection were more affected by the COVID-19 pandemic than those who did not have COVID-19 infection in terms of family members who got COVID-19 or died of COVID-19; school, social, and parent problems; more expenses; and less income, according to this study.

COVID-19-positive children had a higher rate of clinical ratings for affective disorders, anxiety difficulties, pervasive developmental problems, and oppositional defiant problems than COVID-19-negative children in this study.

In the current study, somatic problems had a positive correlation with school problems, social problems, and decrease family income due to COVID-19. Sleep problems had a negative correlation with a number of children. Regarding the CBCL profile of DSM-5 scales, anxiety problems a had positive correlation with school problems, social problems, increase family expenses, and decrease family income due to COVID-19. Having a dead family member from COVID-19 infection had a positive correlation with attention problems and pervasive developmental problems. The total score of the socioeconomic scale had a negative correlation with affective problems.

The first study has examined quarantined children and adolescents and discovered that they were suffering from depression, anxiety, or both. In contrast, the second study was conducted during the pandemic and discovered symptoms of inattention, clinging, worry, and anger [17, 18]. Another study in India has examined quarantined children and adolescents and found a high rate of psychological distress associated with feelings of helplessness, worry, and fear [19].

Another study has evaluated the effects of COVID-19 infection on children aged 6–12 years. It was found that children infected with COVID-19 were more likely to experience withdrawal, anxiety/depression, somatic difficulties, internalizing problems, externalizing problems, and total problems [20].

A significant restriction in social relations to close family members was observed. Contact with peers has been forbidden or severely restricted in various countries [21]. The significance of peer contact in the well-being of kids and adolescents is of great importance as restrictions in peer contact could have a negative impact [22, 23].

In terms of education, numerous countries have experienced school lockdown [24]. As a result, significant negative consequences, such as wasted educational time, limited peer connection, and a lack of daily regularity, must be addressed. Furthermore, in some societies, stigmatization of diseased individuals is common.

The pandemic has caused a reorganization of daily life at the household level. All family members must deal with the strain of social exclusion. Academic disruptions have resulted in homeschooling and the possible postponing of tests. Parents have faced growing pressure to maintain work and enterprises at home and educate school-aged children at home. Caregivers’ assets, such as relatives, have been curtailed, causing disrupted family relationships. The worry of losing a member of a high-risk group’s family can escalate. The pandemic alters families’ regular grief rituals in the event of death. Grief for lost family members, particularly when engagement with an infected individual is limited or denied, can result in adjustment problems, post-traumatic stress disorder, depression, and even suicide in people of all ages [25]. Moreover, parents must explain and describe the COVID-19 outbreak to their children and cope with the concern and stress that come with such uncertain times. COVID-19 may create anxiety in all family members. When all of this occurs simultaneously, it can produce a great deal of stress and psychological distress for the entire family.

The pandemic has significant economic consequences and places financial strain on many households. Economic pressure, especially with social exclusion, has been shown to be associated with a major issue in mental health. Economic recessions, as well as associated issues, such as unemployment, financial difficulty, and unmanageable debts, have been connected to impaired mental health [26, 27]. Parental mental illness and substance addiction have a major impact on parent–child relationships and raise the probability of mental health disorders in children [28, 29].

During the COVID-19 pandemic, a large surge in domestic violence was observed [30]. Financial issues and loss of income can lead to feelings of financial strain and subsequently marital discord [31]. The quarantine can lead to a loss of liberty and privacy and an increase in stress levels. There is the potential for abusers to become more controlling as they take back control. Victims’ chances of escaping abusive partners are reduced, while their exposure to offenders is raised [32]. During the COVID-19 pandemic, an increase in domestic violence was observed worldwide [33]. António Guterres, the UN Secretary General, called attention to a “horrifying global spike in domestic abuse” [34]. Domestic violence has a major impact on children’s mental health [35] and could have long-term implications [36].

The impact of the COVID-19 pandemic on the lives of children and adolescents has been documented in the literature. The COVID-19 pandemic distress disrupted the hypothalamic–pituitary–adrenal axis, which can interfere with various physiological processes during the early stages of development, by increasing the generation and release of inflammatory mediators. This unbalance could result in immune, endocrine, and neurological system dysfunctions and a higher chance of psychiatric diseases later in life [37].

This study provided a few key policy implications for policymakers, which could be used to increase support to families with COVID-19 infection, because infected children suffer more and are more likely to develop mental health problems. As a result, mental screening for youngsters has become more necessary. Psychoeducational programs are needed to make people at school, at home, and in the community aware of these kids.

This study had some problems, for example, its small sample size. So, future studies with large, representative samples will be needed to ensure that our findings are valid. Moreover, we could not examine the association between the severity of COVID-19 infection according to the World Health Organization guidelines [38] and psychological difficulties because most children received care at home. Finally, when compared to adults, children have a greater capability for brain plasticity, particularly in their ability to recover from brain damage or major surgery such as hemispherectomy for epilepsy. Basic mechanisms that promote plasticity during development include neurogenesis persistence in some areas of the brain, neuronal elimination by apoptosis or programmed cell death, postnatal proliferation and synaptic pruning, and activity-dependent refinement of neural connections (Johnston, 2004). So, the long-time consequence of COVID-19 is considered crucial to evaluate brain changes. Also, determining causal relationships between factors was also difficult because of the study’s cross-sectional design. As a result, a longitudinal study should be conducted.

## Conclusions

COVID-19 was found in 21.7% of the children who participated in this study. Children infected with COVID-19 were more likely to have psychological issues, such as affective disorders, anxiety problems, pervasive developmental problems, and oppositional defiant problems.

## Data Availability

All data generated or analyzed during this study are available from corresponded on request.

## Abbreviations

COVID-19: Coronavirus disease 2019
IQ: Intelligence quotient
CBCL: The Checklist for Children’s Behaviour
SES: Socioeconomic status
